# Association between patterns of alcohol consumption (beverage type, frequency and consumption with food) and risk of adverse health outcomes: a prospective cohort study

**DOI:** 10.1186/s12916-020-01878-2

**Published:** 2021-01-12

**Authors:** Bhautesh Dinesh Jani, Ross McQueenie, Barbara I. Nicholl, Ryan Field, Peter Hanlon, Katie I. Gallacher, Frances S. Mair, Jim Lewsey

**Affiliations:** 1 General Practice and Primary Care, Institute of Health and Wellbeing, University of Glasgow, 1 Horselethill Road, Glasgow, G12 9LX UK; 2 Health Economics and Health Technology Assessment, Institute of Health and Wellbeing, University of Glasgow, Glasgow, UK

**Keywords:** Alcoholism, Alcohol drinking, Mortality, Myocardial infarction, Stroke, Liver cirrhosis, Neoplasms, Accidents

## Abstract

**Background:**

Alcohol consumption is a leading contributor to death and disability worldwide, but previous research has not examined the effects of different patterns of alcohol consumption. The study objective was to understand the relationship between different alcohol consumption patterns and adverse health outcomes risk, adjusting for average amount consumed among regular drinkers.

**Methods:**

This was a prospective cohort study of UK Biobank (UKB) participants**.** Abstainers, infrequent alcohol consumers or those with previous cancer, myocardial infarction (MI), stroke or liver cirrhosis were excluded. We used beverage type, consumption with food and consumption frequency as exposures and adjusted for potential confounding. All-cause mortality, major cardiovascular events-MACE (MI/stroke/cardiovascular death), accidents/injuries, liver cirrhosis, all-cause and alcohol-related cancer incidence over 9-year median follow-up period were outcomes of interest.

**Results:**

The final sample size for analysis was *N* = 309,123 (61.5% of UKB sample). Spirit drinking was associated with higher adjusted mortality (hazard ratio (HR) 1.25; 95% confidence intervals (CI) 1.14–1.38), MACE (HR 1.31; 95% CI 1.15–1.50), cirrhosis (HR 1.48; 95% CI 1.08–2.03) and accident/injuries (HR 1.10; 95% CI 1.03–1.19) risk compared to red wine drinking, after adjusting for the average weekly alcohol consumption amounts. Beer/cider drinkers were also at a higher risk of mortality (HR 1.18; 95% CI 1.10–1.27), MACE (HR 1.16; 95% CI 1.05–1.27), cirrhosis (HR 1.36; 95% CI 1.06–1.74) and accidents/injuries (HR 1.11; 95% CI 1.06–1.17). Alcohol consumption without food was associated with higher adjusted mortality (HR 1.10; 95% CI 1.02–1.17) risk, compared to consumption with food. Alcohol consumption over 1–2 times/week had higher adjusted mortality (HR 1.09; 95% CI 1.03–1.16) and MACE (HR 1.14; 95% CI 1.06–1.23) risk, compared to 3–4 times/week, adjusting for the amount of alcohol consumed.

**Conclusion:**

Red wine drinking, consumption with food and spreading alcohol intake over 3–4 days were associated with lower risk of mortality and vascular events among regular alcohol drinkers, after adjusting for the effects of average amount consumed. Selection bias and residual confounding are important possible limitations. These findings, if replicated and validated, have the potential to influence policy and practice advice on less harmful patterns of alcohol consumption.

**Supplementary information:**

The online version contains supplementary material available at 10.1186/s12916-020-01878-2.

## Background

Alcohol consumption is one of the leading risk factors for death and disability-adjusted life years (DALY) globally, based on findings from the recent Global Burden of Disease Study that included data from 195 countries [1]. Heavy alcohol consumption is associated with a higher risk of a number of adverse health outcomes, including all-cause mortality, cancer, cardiovascular events and injuries [1–4]. A review of 255 systematic reviews and meta-analyses has suggested the need for further research into the health risks associated with different patterns of alcohol consumption (beyond average amount), such as frequency of consumption, consumption of alcohol with or without food and type of alcoholic beverage [2]. Most guidelines on safe/risky levels of alcohol consumption focus on average daily or weekly amount of alcohol; however, recommendations on the pattern or type of alcohol are not available [5]. For example, the UK Chief Medical Officer has advised not to regularly consume more than 14 units (112 g) of alcohol per week and to spread alcohol consumption evenly over 3 or more days in a week [6], without any specific advice on the type of alcohol or any suggestions on the timing of consuming alcohol in relation to eating food.

There is some evidence to suggest that other dimensions of alcohol consumption beyond the amount of alcohol may influence the risk of adverse health outcomes. A recent study on the frequency of alcohol consumption in > 430,000 participants from two independent US datasets from a community and an outpatient population, respectively, found that there was a higher risk of all-cause and cardiovascular mortality among binge drinkers (1–2 days/week) and very frequent drinkers (6–7 days/week) compared to those who consumed alcohol in moderate amounts over 3 days/week [7]. Previous research has suggested that wine drinking (compared to beer or spirit drinking) is associated with lower risk of all-cause mortality [3, 8, 9] and cardiovascular events [3, 8, 10]. In an Italian cohort, wine drinking with food was associated with lower all-cause mortality and cardiovascular events compared to those drinking wine without food [11]. A recently published study on approximately 400,000 UK women suggested that for a given amount of alcohol consumption, daily alcohol consumption and consumption without food were associated with higher risk of liver cirrhosis [12].

However, none of the previous research studies have comprehensively investigated different dimensions of alcohol consumption patterns together and its potential effects on risk of adverse health outcomes. Secondly, most previous research has used non-drinkers as the reference category which makes the direct comparison of different patterns of alcohol consumption on health outcomes difficult. Hence, there is currently a lack of evidence to support recommendations about the frequency of alcohol consumption, whether alcohol is best consumed with or without food, and the type of alcoholic beverage.

The aim of this study is to examine the combined effect of various patterns of alcohol consumption on risk of adverse health outcomes among regular alcohol drinkers, adjusting for average amount of weekly alcohol consumption.

## Methods

### Study design and participants

UK Biobank is a prospective population-based cohort study of 502,616 participants enrolled from 22 different assessment centres across England, Scotland and Wales between 2006 and 2010 (5% response rate). Individuals were invited to participate on a voluntary basis if they lived within 25 miles of a UK Biobank assessment centre and were registered with a General Practitioner; all participants gave informed consent for data provision and linkage. UK Biobank has full ethical approval from the NHS National Research Ethics Service (16/NW/0274). A detailed account of alcohol consumption patterns, sociodemographic, lifestyle and medical information was collected from all participants recruited to the study.

### Information on alcohol consumption

Participants completed a touchscreen questionnaire to report their frequency of alcohol intake, average amount and type of alcoholic beverage and how they consume alcohol in relation to food.  Participants who abstained from alcohol (due to various reasons https://biobank.ctsu.ox.ac.uk/crystal/field.cgi?id=3859) and those with missing values were excluded from the analysis. Participants who reported drinking alcohol infrequently (e.g. special occasions and one to three times a month) were also excluded from analysis, as although they did report their average amount of alcohol consumed, the purpose of this analysis was to study the health risks associated with different drinking patterns among regular alcohol drinkers. Average weekly intake of red wine, spirits, beer plus cider, champagne plus white wine, fortified wine and other alcoholic drinks was reported at the time of study recruitment. Using this information, we calculated the total average weekly units of alcohol. If red wine consumption accounted for more than 50% of the total weekly units consumed by a participant on average, then that participant was labelled as “red wine drinker”. Using similar definitions, type of beverage was classified into five categories: red wine, beer/cider, spirits, white wine/fortified wine/champagne and mixed. Participants were asked if they usually drink alcohol with food and based on their answers classified into yes, no and mixed. The frequency of alcohol intake over the week was divided into three categories: daily or almost daily, three to four times a week and once or twice a week. Average weekly alcohol units was used as a continuous variable and also divided into the following categories for sensitivity analysis: 1–14 units (low risk), 15–35 units in females and 15–50 units in males (increasing risk) and > 35 units in females and > 50 units in males (higher risk), adapted from the latest health survey in England and from information incorporated in NICE guidelines [13].

### Demographics, lifestyle, biomarkers and long-term conditions (LTCs) information

Socioeconomic status was classified based on Townsend score (a measure of deprivation in the UK) [14]. A Townsend deprivation score calculated using the participant’s home postcode, based on the preceding national census output areas, was provided; a higher score implied higher levels of socioeconomic deprivation. Townsend score was divided into five quintiles. Smoking status was divided into three categories: non-smokers, previous smokers and current smokers. Physical activity was self-reported and classified as: none (no physical activity in the last 4 weeks), low (light activity only in the last 4 weeks), medium (heavy walking for pleasure and/or other exercises in the last 4 weeks) and high (strenuous sports in the last 4 weeks) [15, 16]. Body mass index (BMI) calculated from anthropometric measurements at the baseline assessment was classified as per WHO classification into < 18.5, 18.5–24.9, 25–29.9, 30–34.9, 35–39.9 and ≥ 40 kg/m^2^ [17]. Systolic blood pressure was recorded using an automated machine by two readings at baseline and classified into < 120, 120–139, 140–159 and > 160 mmHg [18]. Total cholesterol levels were measured at baseline and categorised into ≥ 5.0 mmol/L and < 5.0 mmol/L [19]. C-reactive protein levels (in mg/L) and gamma glutamyltransferase levels (in U/L) were measured at baseline and used as continuous variables. Self-rated health was classified into excellent, good, fair and poor by participants at baseline. The physical and mental health conditions (including diabetes and hypertension) self-reported by participants were organised into a list of 42 long-term conditions (LTCs) based on our previously published literature on multimorbidity (see supplementary Table S1) [20, 21]. The number of LTCs was classified based on LTC count into 0 LTCs, 1 LTC, 2 LTCs, 3 LTCs and ≥ 4 LTCs.

### Clinical outcomes

The baseline assessment centre data were linked to national mortality, cancer and hospital episode statistics records by UK Biobank data analysts. The six outcomes studied were all-cause mortality, major adverse cardiovascular event-MACE (stroke, myocardial infarction (MI) or vascular death), external causes of injuries/accidents, incidence of all-cause and alcohol-related cancers (colon, rectum, breast, liver, oesophagus and larynx) [22]. Participants with previous history of stroke, MI, liver cirrhosis or any cancer were removed from analysis to avoid reverse causality. All-cause mortality, stroke, MI and cancer incidence events were reported by UK Biobank data analysis team through data linkage. We utilised ICD-10 classifications for defining vascular deaths (ICD-10 codes “I00-I78”, “G45” and “G46” as primary cause of death), liver cirrhosis (hospitalisation events with primary diagnostic ICD-10 codes “K70” and “K74”) and external causes of injuries/accidents (hospitalisation events with primary diagnostic ICD-10 codes beginning with “W”, “X”, “V” and “Y0”) [23]. The follow-up period ended between November 2015 and January 2016, depending on different assessment centres across the UK. Length of follow-up was a median duration of 9 years (Interquartile range 8.3–9.5 years).

### Statistical analysis

The distribution of various demographic, health-related behaviour characteristics, frequency of alcohol consumption, type of alcoholic beverages consumed and alcohol consumption with/without food was described across the three levels of average alcoholic weekly units (low risk, increasing risk, high risk), using mean and standard deviation for continuous variables and percentages for categorical variables. Six different Cox’s proportional hazards regression models [24] were fitted for the six clinical outcomes under consideration: all-cause mortality, MACE, liver cirrhosis, injuries/accidents, incidence of all-cause and alcohol-related cancers using the final study sample, after all exclusions. In each of these models, age was used as the underlying time variable [25]. Three patterns of alcohol consumption were used together as predictor variables: frequency of alcohol consumption, type of alcoholic beverage and alcohol consumption with food. Results were presented as hazard ratios (HR) with 95% confidence intervals (CI), adjusted for confounding variables (average weekly alcohol units-continuous, sex, socioeconomic status (based on Townsend score), smoking, physical activity levels, BMI and number of LTCs). The models for MACE were adjusted for presence of diabetes, hypertension, systolic blood pressure and total cholesterol values at baseline, in addition to the confounders listed above as they have been recognised as cardiovascular risk factors by the WHO [26]. The total number of participants included in the survival analysis models varied according to the completeness of the putative confounding variables and all missing data were excluded from regression modelling. In view of a large number of co-variates, a global *p* value for heterogeneity was calculated using the “globaltest” package for each regression model, respectively [27].

Marginal fractional polynomials [28] were used to visualise the relationship between average weekly alcohol units (continuous) and two outcomes of interest (all-cause mortality and MACE). In the next step, a set of predicted probability values for the outcome of interest (all-cause mortality and MACE at 7 years minimum duration of follow-up) were calculated from the Cox regression models described above using multiple fractional polynomials for weekly alcohol units and marginal standardisation in which predicted probabilities of the outcome were calculated for every observed confounder value (each category for categorical covariate and mean value for continuous covariates) [29]. The results were visualised by plotting 7-year predicted probability of all-cause mortality and MACE, respectively, against weekly alcohol units, using sub-groups based on three patterns of alcohol consumption with three different sub-plots [30].

### Mediation analysis

The mediating effects of five variables on the relationship between alcohol consumption patterns and clinical outcomes (all-cause mortality and MACE) were examined: amount of average weekly alcohol units (continuous), socio-economic status using Townsend score (continuous), CRP levels (continuous), smoking status (categorical) and self-rated health (categorical). The outcomes of interest (all-cause mortality and MACE) were regressed by the primary exposure variable (alcohol consumption pattern) and all other covariates as per the main analysis. The potential mediators, as listed above, were then regressed by primary exposure variable and all other covariates. The results of the outcome and mediator models were combined to estimate the proportion of average mediated effect and 95% confidence intervals were calculated using Quasi-Bayesian estimates with 100 iterations. This analysis was performed using the “mediation package” [31].

### Sensitivity analyses

The above analysis was repeated using a different classification for type of alcoholic beverage where the classification was based on the drink type consumed in volumes larger than any other drink type by a participant from the total weekly alcohol units instead of > 50% of the total. In addition, several other sensitivity analyses were performed with the entire analyses repeated in sub-groups stratified based on amount of average alcoholic weekly units (low risk, increasing risk, high risk) and sex (male and female), after excluding participants with poor self-rated health at baseline and after excluding first 2 years of follow-up to mitigate the impact of reverse causality.

### Repeated measurement of alcohol consumption

A small number of participants, selected at random for imaging study, self-reported their alcohol intake and consumption pattern during the follow-up period. This information was captured. Average weekly alcohol units and alcohol consumption pattern were classified using the same methods described above. Changes in amount and alcohol consumption pattern were reported using percentages. To model the repeat measurements of alcohol consumption patterns with the outcomes of interest, an extended Cox’s proportional hazards models were fit which allowed for time-varying exposure variables.

All statistical analyses were conducted using R version R-3.6.1.

## Results

### Participant characteristics and alcohol consumption patterns

**Fig. 1:**
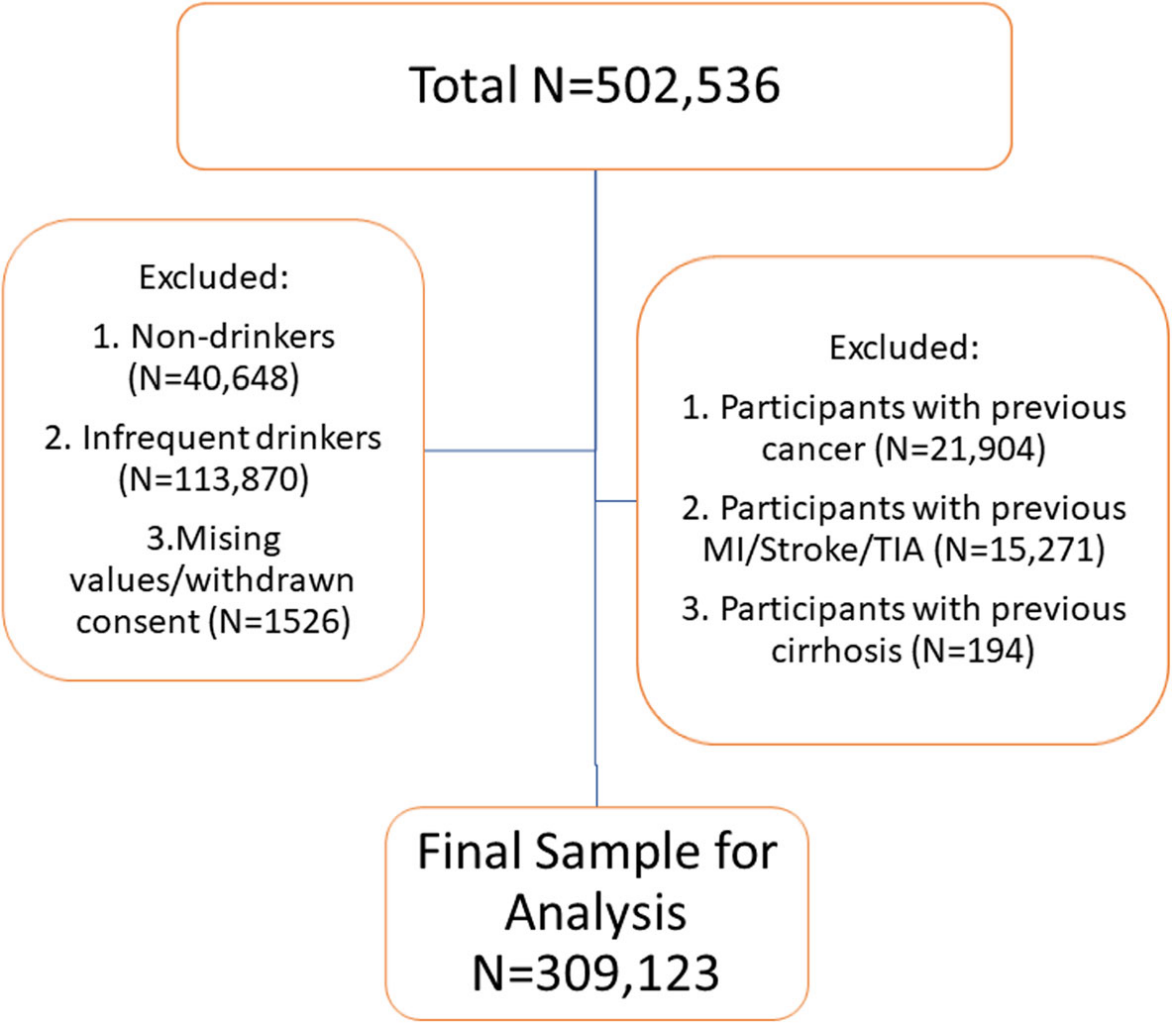
Study sample size: weekly alcohol drinkers among UK Biobank sample. Infrequent drinkers = those reported drinking alcohol on special occasions only or with a frequency of 1–3 times/month. MI, myocardial infarction; TIA, transient ischaemic attack

From the total UK Biobank sample (*N* = 502,536), participants who identified themselves as never consuming alcohol (*N* = 40,648), or consuming alcohol on an infrequent basis on special occasions or with a frequency of 1–3 times/month (*N* = 113,870), were excluded from the analysis. Participants with a previous history of previous cancer, stroke or TIA based on self-report or their electronic health records and participants who withdrew consent for follow-up were also excluded from the analysis (Fig. 1). The final sample size for analysis was *N* = 309,123 (61.5% of the total UK Biobank sample). The age range for the participants in the study sample was from 38 to 73 years.

Less than half of the regular alcohol drinkers (*N* = 147,769; 47.8%) reported consuming 1–14 average weekly units, the recommended amount for low-risk alcohol consumption. A total of *N* = 133,910 (43.3% of regular alcohol drinkers) participants reported consuming alcohol in amounts regarded as increasing risk (15–35 average weekly units in females; 15–50 average weekly units in males). A total of *N* = 27,444 (8.9% of regular alcohol drinkers) participants reported drinking high risk (> 35 average weekly units in females; > 50 average weekly units in males) amounts of alcohol. Table 1 shows the distribution of demographics and lifestyle factors, frequency, type and pattern of alcohol consumption, among the different groups of regular alcohol drinkers, based on average weekly units consumed.

**Table 1:** Distribution of demographics, lifestyle, biomarkers and alcohol drinking patterns across different amounts of average weekly alcohol consumption

	Categories	Low risk *N* = 147,769 (47.8%)	Increasing risk *N* = 133,910 (43.3%)	Higher risk *N* = 27,444 (8.9%)	Total sample *N* = 309,123
Age in years (mean); missing values = 0		56.4	55.9	55.3	56.1
Sex; missing values = 0	Females	101,067 (65.3%)	46,463 (30%)	7152 (4.6%)	154,682 (100%)
	Males	46,702 (30.3%)	87,447 (56.6%)	20,292 (13.1%)	154,441 (100%)
Smoking status; missing values = 974 (0.3%)	Never	92,419 (56.9%)	61,711 (38%)	8310 (5.1%)	162,440 (100%)
	Previous	45,560 (39.9%)	56,100 (49.2%)	12,508 (10.9%)	114,168 (100%)
	Current	9314 (29.5%)	15,680 (49.7%)	6547 (20.8%)	31,541 (100%)
Townsend score quintiles; missing values = 360 (0.1%)	0–20 (most affluent)	34,658 (50.9%)	28,908 (42.4%)	4559 (6.7%)	68,125 (100%)
	80–100 (most deprived)	21,586 (42.3%)	22,727 (44.5%)	6734 (13.2%)	51,047 (100%)
Number of LTCs; missing values = 5 (< 0.1%)	0	54,566 (48.5%)	50,048 (44.2%)	8635 (7.6%)	113,249 (100%)
	4 or more	7581 (48.6%)	6217 (39.8%)	1818 (11.6%)	15,616 (100%)
Body mass index (mean); missing values = 1237 (0.4%)		26.6	27.2	27.9	27
C-reactive protein (mean); missing values = 19,688 (6.3%)		2.31	2.30	2.76	2.34
Gamma glutamyl transferase (mean); missing values = 19,260 (6.2%)		31.4	40.4	65	38.3
Physical activity levels; missing values = 2987 (1%)	High	15,234 (40.4%)	19,298 (51.2%)	3169 (8.4%)	37,701 (100%)
	None	6629 (46.%)	5470 (38.3%)	2170 (15.2%)	14,269 (100%)
Systolic blood pressure; missing values = 9644 (3.1%)	< 120	26,476 (60%)	15,653 (35.5%)	1977 (4.5%)	44,106 (100%)
	120–139	59,707 (48.8%)	53,514 (43.7%)	9174 (7.5%)	122,395 (100%)
	140–159	40,862 (43.4%)	43,191 (45.8%)	10,174 (10.8%)	94,227 (100%)
	> 160	16,130 (41.6%)	17,421 (44.9%)	5200 (13.5%)	38,751 (100%)
Total cholesterol; missing values = 19,107 (6.1%)	< 5.0	29,579 (47.8%)	27,480 (44.3%)	4912 (7.9%)	61,971 (100%)
	5.0 or more	108,927 (47.8%)	98,334 (43.1%)	20,784 (9.1%)	228,045 (100%)
Self-rated health; missing values = 907 (0.3%)	Excellent	29,554 (50.1%)	25,731 (43.6%)	3702 (6.3%)	58,987 (100%)
	Poor	3083 (41%)	3035 (40.4%)	1398 (18.6%)	7516 (100%)
Alcohol consumed with food; missing values = 0	Yes	78,466 (58.8%)	49,946 (37.4%)	5142 (3.8%)	133,554 (100%)
	No	23,823 (37.5%)	29,576 (46.6%)	10,119 (15.9%)	63,518 (100%)
	Mixed	45,490 (40.6%)	54,397 (48.5%)	12,185 (10.9%)	112,072 (100%)
Type of alcohol consumed; missing values = 0	Red wine	43,487 (53.9%)	32,842 (40.7%)	4333 (5.4%)	80,662 (100%)
	Beer or cider	24,682 (27.7%)	49,247 (55.2%)	15,218 (17.1%)	89,147 (100%)
	White/sparkling wine	32,407 (59.6%)	18,759 (34.5%)	3192 (5.9%)	54,358 (100%)
	Spirits	10,241 (62.5%)	5071 (30.9%)	1085 (6.6%)	16,397 (100%)
	Others/mixed	36,962 (53.9%)	28,000 (40.8%)	3618 (5.3%)	68,580 (100%)
Frequency of alcohol consumption; missing values = 0	3–4 times a week	43,158 (41.6%)	54,576 (52.6%)	5991 (5.8%)	103,725 (100%)
	1–2 times a week	89,173 (77.4%)	25,349 (22%)	755 (0.6%)	115,277 (100%)
	Daily or almost daily	15,448 (17.1%)	53,994 (59.9%)	20,700 (23%)	90,142 (100%)

Legend: Townsend score is a measure of socio-economic status based on participant’s home postcode. LTCs = long-term conditions. Low risk: 1–14 average weekly alcohol units; increasing risk: 15–35 (females) and 15–50 (males) average weekly alcohol units; higher risk: > 35 (females) and > 50 (males) average weekly alcohol unit

Table 1 shows that increasing and higher risk amounts of weekly alcohol consumption were proportionately more common among males, former/current smokers and those from deprived socio-economic background. Participants who reported drinking alcohol once or twice in a week and consuming alcohol with food had proportionately higher number of people with low-risk weekly alcohol consumption amount than those who reported consuming alcohol more frequently and without food. Alcohol consumption in higher risk amounts was proportionately much higher in predominantly beer or cider drinkers, when compared to all other types of alcoholic beverages. The median duration of follow-up was 9 years (interquartile range 8.3–9.5 years). At the end of the follow-up period, 8869 participants were dead (2.9%), 5246 participants had experienced a MACE event (1.7%), 838 developed liver cirrhosis (0.3%) and 16,818 (5.4%) were noted to have accidents/self-harm, while the incidence of all-cause cancer was 27,543 (8.9%) and of alcohol-related cancer was 6529 (2.1%).

### Type of alcohol and health risk

**Fig. 2:**
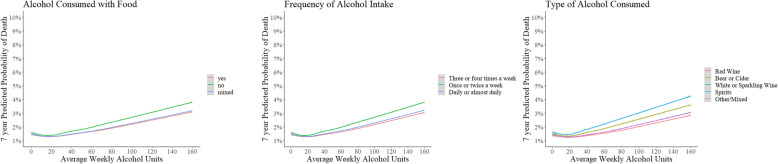
Predicted probability (7-year) of mortality, average amount of total weekly alcohol units and different patterns of alcohol consumption. All results adjusted for age, sex, Townsend score for socio-economic deprivation, smoking habits, BMI, physical activity levels, number of long-term conditions, self-rated health and CRP levels

**Fig. 3:**
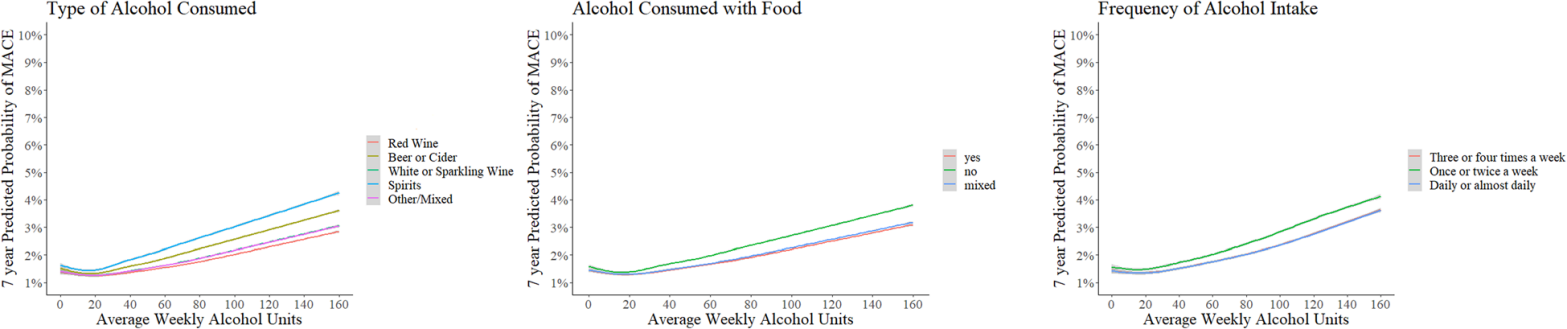
Predicted probability (7-year) of major adverse cardiovascular event-MACE (myocardial infarction/stroke/cardiovascular death), average amount of total weekly alcohol units and different patterns of alcohol consumption. All results adjusted for age, sex, Townsend score for socio-economic deprivation, smoking habits, BMI, physical activity levels, number of long-term conditions, self -rated health, CRP levels, systolic blood pressure, total cholesterol levels, diabetes and hypertension

Table 2 shows the results from Cox’s survival analyses (with age included as time variable) comparing risk for various poor health outcomes for different types of alcohol. All models were also adjusted for average weekly amount of alcohol in weekly units, sex, socio-economic status, smoking habits, BMI, physical activity levels, number of LTCs, self-rated health and CRP levels. The models for MACE were additionally adjusted for presence of systolic blood pressure, total cholesterol levels, diabetes and hypertension. The absolute event rates for all-cause mortality and MACE were found to be highest among predominantly spirit drinkers, followed by predominantly beer/cider drinkers (see Table 2). Participants who consumed predominantly spirits were found to have a significantly higher adjusted relative risk of all-cause mortality (hazard ratio (HR) = 1.25; 95% confidence intervals (CI) 1.14 to 1.38), MACE (HR = 1.31; 95% CI 1.15 to 1.50), liver cirrhosis (HR = 1.48; 95% CI 1.08 to 2.03) and accidents/self-harm (HR 1.10; 95% CI 1.03 to 1.19), when compared to participants who consumed predominantly red wine. Beer/cider drinkers were also found to have a significantly higher relative risk of all-cause mortality (HR = 1.18; 95% CI 1.10 to 1.27), MACE (HR 1.16; 95% CI 1.05 to 1.27), liver cirrhosis (HR 1.36; 95% CI 1.06 to 1.74) and accidents/self-harm (HR = 1.11; 95% CI 1.06 to 1.17), compared to red wine drinking counterparts. Type of alcoholic beverage did not have an association with the risk of all-cause cancer and alcohol-related cancer incidence. The adjusted 7-year predicted probability of dying and experiencing a MACE event was lowest among predominantly red wine drinkers, across the whole spectrum of weekly alcohol consumption amounts (Figs. 2 and 3 respectively).

**Table 2:** Type of alcohol consumed and adjusted risk for poor health outcomes

N = 309,123. Number used for analysis. *N* = 286,115 (92.6%) used for analysis after excluding missing values.												
Type of alcohol consumed	All-cause mortality		MACE		Liver cirrhosis		Accidents/self-harm/assaults		New cancer (all-cause) incidence		Alcohol cancer incidence	
	Events = 8869 (2.9%)	HR with 95% CI; *p* value	Events = 5246 (1.7%)	HR with 95% CI; *p* value	Events = 838 (0.3%)	HR with 95% CI; *p* value	Events = 16,818 (5.4%)	HR with 95% CI; *p* value	Events = 27,543 (8.9%)	HR with 95% CI; *p* value	Events = 6529 (2.1%)	HR with 95% CI; *p* value
Red wine drinkers (reference) *N* = 80,655 (26.1%)	1847 (2.3%)	1	1067 (1.3%)	1	119 (0.1%)	1	4090 (5.1%)	1	7192 (8.9%)	1	1878 (2.3%)	1
Beer or cider drinkers *N* = 89,139 (28.9%)	3441 (3.8%)	1.18 (1.10–1.27); p < 0.01	2076 (2.3%)	1.16 (1.05–1.27); *p* < 0.01	401 (0.4%)	1.36 (1.06–1.74); *p* = 0.02	5290 (5.9%)	1.11 (1.06–1.17); *p* < 0.01	7950 (8.9%)	0.99 (0.95–1.03); *p* = 0.56	1294 (1.4%)	1.02 (0.94–1.12); *p* = 0.59
White wine drinkers *N* = 54,355 (17.5%)	1121 (2.1%)	1.03 (0.95–1.11); *p* = 0.49	606 (1.1%)	1.05 (0.94–1.18); *p* = 0.33	105 (0.2%)	1.21 (0.91–1.61); *p* = 0.19	2885 (5.3%)	1.03 (0.98–1.09); *p* = 0.19	4483 (8.2%)	1.03 (0.99–1.07); *p* = 0.19	1489 (2.7%)	1.02 (0.95–1.10); *p* = 0.53
Spirits drinkers *N* = 16,397 (5.3%)	717 (4.4%)	1.25 (1.14–1.38); p < 0.01	396 (2.4%)	1.31 (1.15–1.50); *p* < 0.01	92 (0.6%)	1.48 (1.08–2.03); *p* = 0.01	1086 (6.6%)	1.10 (1.03–1.19); p < 0.01	1581 (9.6%)	1.00 (0.95–1.07); *p* = 0.85	435 (2.7%)	1.03 (0.91–1.15); *p* = 0.65
Mixed *N* = 68,577 (22.2%)	1743 (2.5%)	1.08 (1.01–1.16); *p* = 0.03	1101 (1.6%)	1.16 (1.06–1.27); p = 0.01	121 (0.2%)	1.14 (0.87–1.48); p = 0.19	3467 (5.1%)	1.02 (0.97–1.07); *p* = 0.41	6337 (9.2%)	1.03 (0.99–1.07); *p* = 0.08	1433 (2.1%)	1.00 (0.93–1.08); *p* = 0.96
Global *p* value for heterogeneity	–	< 0.01	–	< 0.01	–	< 0.01	–	< 0.01	–	< 0.01	–	0.03

Legend: *HR* hazard Ratio, *CI* confidence intervals, *MACE* major adverse cardiovascular event. Alcohol new cancers = breast, colon, rectum, larynx, liver and oesophagus. All results adjusted for age, sex, Townsend score for socio-economic deprivation (continuous), average weekly alcohol units (continuous), alcohol consumption frequency, alcohol consumption pattern with/without meals, smoking habits, BMI, physical activity levels, number of long-term conditions, self-rated health and C-reactive protein levels at baseline. MACE events model adjusted for all the above plus presence of diabetes, hypertension, systolic blood pressure and total cholesterol levels at baseline. Cirrhosis events model adjusted for all of the above plus gamma glutamyl transpeptidase levels at baseline

### Alcohol consumption with/without food and health risk

The absolute event rate for death, MACE and accidents/self-harm was higher among participants drinking alcohol without food (see Table 3). The adjusted relative risk of all-cause mortality (HR = 1.10, 95% CI 1.02 to 1.17) was higher among participants consuming alcohol without food compared to participants consuming alcohol with food. Consumption pattern of drinking alcohol with or without food did not have an association with the risk of MACE, liver cirrhosis, accidents/self-harm, all-cause cancer and alcohol-related cancer incidence. The 7-year predicted probability of dying and experiencing a MACE event was higher for participants drinking alcohol without food, across the whole spectrum of weekly alcohol consumption amounts (Figs. 2 and 3, respectively).

**Table 3:** Alcohol consumption with or without food and adjusted risk for poor clinical outcomes, stratified sub-groups based on average alcohol consumption

N = 309,123. Number used for analysis *N* = 286,115 (92.6%) used for analysis after excluding missing values.												
Alcohol consumed with/without food	All-cause mortality		MACE		Liver cirrhosis		Accidents/self-harm/assaults		New cancer (all-cause) incidence		Alcohol cancer incidence	
	Events = 8869 (2.9%)	HR with 95% CI; *p* value	Events = 5246 (1.7%)	HR with 95% CI; *p* value	Events = 838 (0.3%)	HR with 95% CI; *p* value	Events = 16,818 (5.4%)	HR with 95% CI; *p* value	Events = 27,543 (8.9%)	HR with 95% CI; *p* value	Events = 6529 (2.1%)	HR with 95% CI; *p* value
Yes (reference) *N* = 133,545 (43.2%)	3289 (2.5%)	1	1937 (1.4%)	1	228 (0.2%)	1	6819 (5.1%)	1	12,441 (9.3%)	1	3100 (2.3%)	1
No *N* = 63,514 (20.6%)	2603 (4.1%)	1.10 (1.02–1.17); p = 0.01	1482 (2.3%)	1.08 (0.99–1.17); *p* = 0.09	304 (0.5%)	0.88 (0.71–1.11); *p* = 0.29	4004 (6.3%)	1.03 (0.98–1.08); *p* = 041	5551 (8.7%)	0.96 (0.92–1.00); *p* = 0.05	1173 (1.8%)	0.94 (0.87–1.02); *p* = 0.14
Mixed *N* = 112,064 (36.2%)	2977 (2.7%)	1.01 (0.96–1.07); *p* = 0.75	1827 (1.6%)	1.01 (0.94–1.09); *p* = 0.77	306 (0.3%)	1.00 (0.82–1.22); p = 0.96	5995 (5.3%)	1.00 (0.97–1.04); *p* = 0.82	9551 (8.5%)	1.00 (0.97–1.03); p = 0.96	2256 (2%)	0.98 (0.92–1.04); *p* = 0.50
Global *p* value for heterogeneity	–	< 0.01	–	< 0.01	–	< 0.01	–	< 0.01	–	< 0.01	–	0.03

Legend: *HR* hazard ratio, *CI* confidence intervals, *MACE* major adverse cardiovascular event. Alcohol new cancers = breast, colon, rectum, larynx, liver and oesophagus. All results adjusted for age, sex, Townsend score for socio-economic deprivation (continuous), average weekly alcohol units (continuous), frequency of alcohol consumption, type of alcohol consumed, smoking habits, BMI, physical activity levels, number of long-term conditions, self-rated health and C-reactive protein levels at baseline. MACE events model adjusted for all the above plus presence of diabetes, hypertension, systolic blood pressure and total cholesterol levels at baseline. Cirrhosis events model adjusted for all of the above plus gamma glutamyl transpeptidase levels at baseline

### Alcohol consumption frequency and health risk

Once or twice weekly alcohol consumption was associated with higher adjusted relative risk of mortality (HR = 1.09; 95% CI 1.03–1.16) and MACE (HR = 1.14; 95% CI 1.06–1.23) compared to participants drinking alcohol over three to 4 days in a week (see Table 4), while participants drinking alcohol daily or almost daily were found to have a higher adjusted relative risk of liver cirrhosis (HR = 1.49; 95% CI 1.21 to 1.82). Frequency of alcohol consumption over the week did not have an association with the risk of all-cause cancer and alcohol-related cancer incidence. Participants spreading their alcohol intake over 3 to 4 days in a week had lower predicted probability for mortality and MACE (see Figs. 2 and 3, respectively), and lower corresponding absolute event rates (see Table 4).

**Table 4:** Alcohol consumption frequency and adjusted risk for poor clinical outcomes, stratified sub-groups based on average alcohol consumption

N = 309,123. Number used for analysis *N* = 286,115 (92.6%) used for analysis after excluding missing values												
Type of alcohol consumed	All-cause mortality		MACE		Liver Cirrhosis		Accidents/self-harm/assaults		New cancer (all-cause) incidence		Alcohol cancer incidence	
	Events = 8869 (2.9%)	HR with 95% CI	Events = 5246 (1.7%)	HR with 95% CI	Events = 838 (0.3%)	HR with 95% CI	Events = 16,818 (5.4%)	HR with 95% CI	Events = 27,543 (8.9%)	HR with 95% CI	Events = 6529 (2.1%)	HR with 95% CI
3–4 times/week (reference) *N* = 103,717 (33.5%)	2598 (2.5%)	1	1603 (1.5%)	1	180 (0.2%)	1	5336 (5.1%)	1	8996 (8.7%)	1	2144 (2.1%)	1
1–2 times/week *N* = 115,270 (37.3%)	3084 (2.7%)	1.09 (1.03–1.16); p < 0.01	1921 (1.7%)	1.14 (1.06–1.23); *p* < 0.01	188 (0.2%)	1.04 (0.83–1.31); *p* = 0.69	6153 (5.3%)	1.04 (1.00–1.09); *p* = 0.04	9612 (8.3%)	1.02 (0.99–1.05); *p* = 0.17	2376 (2.1%)	1.01 (0.95–1.08); *p* = 0.73
Daily or almost daily *N* = 90,136 (29.2%)	3187 (3.5%)	1.06 (0.99–1.12); *p* = 0.052	1722 (1.9%)	0.99 (0.91–1.07); *p* = 0.80	470 (0.5%)	1.49 (1.21–1.82); *p* < 0.01	5329 (5.9%)	1.02 (0.97–1.06); *p* = 0.43	8935 (9.9%)	0.99 (0.96–1.02); *p* = 0.65	2009 (2.2%)	0.98 (0.87–1.02); *p* = 0.50
Global *p* value for heterogeneity	–	< 0.01	–	< 0.01	–	< 0.01	–	< 0.01	–	< 0.01	–	0.03

Legend: *HR* hazard ratio, *CI* confidence intervals, *MACE* major adverse cardiovascular event. Alcohol new cancers = breast, colon, rectum, larynx, liver and oesophagus. All results adjusted for age, sex, Townsend score for socio-economic deprivation (continuous), average weekly alcohol units (continuous), type of alcohol consumed, alcohol consumption pattern with/without meals, smoking habits, BMI, physical activity levels, number of long-term conditions,, self-rated health and C-reactive protein levels at baseline. MACE events model adjusted for all the above plus presence of diabetes, hypertension, systolic blood pressure and total cholesterol levels at baseline. Cirrhosis events model adjusted for all of the above plus gamma glutamyl transpeptidase levels at baseline

### Mediation analysis

All five variables (amount of average weekly alcohol units, socio-economic status using Townsend score, CRP levels, smoking status and self-rated health) were found to have some mediating effect on the observed relationship between alcohol consumption pattern and clinical outcomes (all-cause mortality and MACE), with considerable variation in proportion of the effect mediated. The weekly average amount of alcohol consumed was found to have the largest mediation effect sizes (ranging from 3.5 to 19.5%). Please see supplementary Table S2 for the full results.

### Sensitivity analyses

The trends observed in the main analysis were unchanged in sensitivity analyses performed with alternative classification of type of alcoholic beverage (supplementary Table S3), after excluding outcomes for the first 2 years of follow-up (supplementary Table S4), and after excluding participants with poor self-rated health at baseline (supplementary Table S5). In stratified sub-group analysis based on average amount of weekly alcoholic units consumed, beer drinkers were found to have a higher relative risk of mortality and MACE with “low-risk” alcohol consumption, while spirit drinkers were found to have large relative effect sizes on mortality, MACE and cirrhosis with “increasing” and “high-risk” amounts of alcohol consumption (see supplementary Tables S6-S8). Drinking alcohol with a frequency of 1–2 times/week was associated with a higher relative risk of MACE with “increasing” and “high-risk” amounts of alcohol consumption while the daily or almost daily frequency of alcohol consumption was associated with higher relative risk of cirrhosis (across all amounts of alcohol consumption). In stratified analysis based on sex, larger effect sizes on relative risk of mortality, MACE and cirrhosis were observed among spirit drinkers in male sub-group analysis compared to female sub-group analysis (see supplementary Tables S9–10).

### Changes in alcohol consumption pattern during follow-up period

During the follow-up period, 15,750 participants (5.1% of the included analysis sample) had at least one measurement of average weekly amount and consumption pattern for their alcohol intake. Majority of participants with repeated measurement (*N* = 14,155; 90%) reported change in the amount of weekly alcohol units consumed, with average mean change from baseline reported as 1.5 alcohol units less than what they consumed at baseline. Nearly half of the participants (*N* = 7305; 46.3%) reported at least one change in their alcohol consumption pattern from baseline. Supplementary Table S11 reports the number of participants changing alcohol consumption pattern from baseline for each consumption pattern category. Supplementary Table S12 reports the results of extended Cox’s model using alcohol consumption pattern as time-varying exposure variable. The observed effect sizes between alcohol consumption pattern and clinical outcomes of interest were not statistically significant.

## Discussion

In this large study of UK adults who consume alcohol regularly, without history of previous cancer/stroke/MI/cirrhosis, the majority of participants (52.2%) reported drinking greater amounts of alcohol than the recommended amounts for low-risk consumption. The highest absolute and relative risk of mortality, experiencing a major adverse cardiovascular event, liver cirrhosis and accidents/self-harm, was observed among participants who were predominantly drinking spirits, followed by beer/cider drinking, compared to red wine drinking. Similarly, participants who reported drinking alcohol without food were at a higher risk of compared to participants consuming alcohol with food. Finally, consuming average weekly amount of alcohol over 1–2 days in a week had higher relative risk of mortality and cardiovascular events while consuming alcohol daily or almost daily had higher relative risk of developing cirrhosis. These results were adjusted for average amount of weekly alcohol units consumed, demographics, lifestyle factors, number of LTCs, self-rated health and biomarkers.

In our study, we found that spirit drinking was associated with 25% higher risk of mortality, 31% higher risk of MACE, 48% higher risk of liver cirrhosis and 10% higher risk of accidents/self-harm, compared to red wine drinkers. Similarly, beer/cider drinking was associated with approximately 18%, 16%, 36% and 11% higher risk of mortality, MACE, liver cirrhosis and accidents/self-harm respectively. Previous research has suggested higher risk of all-cause mortality among beer and spirit drinking when compared to wine drinking [8, 9] and higher risk of cardiovascular deaths among spirit drinkers [8]. However, a direct comparison of the effect sizes in their findings is not feasible as the reference category used in these studies was different (non-drinkers) from this study (red wine drinkers in similar amount). In our study, we found a higher risk of 10% higher risk of mortality with alcohol drinking without food compared to alcohol drinking with food. In an Italian study, wine drinking without food was found to have a higher mortality risk and wine drinking with meals was found to have a lower mortality risk compared to non-drinkers [11]. However, again a direct comparison of effect sizes with their findings is not feasible due to different reference categories used. We observed that daily/almost daily alcohol consumption was associated with 9% higher mortality risk and once or twice weekly alcohol consumption was associated with 14% higher mortality risk compared to alcohol consumption over three to four times in a week. Hartz and colleagues observed different effect sizes with 26% higher risk of mortality with daily drinking and 4% higher mortality risk with drinking once in a week, compared to drinking alcohol 3.2 times/weekly [7]. We did not find an association between various patterns of alcohol consumption and risk of all-cause and alcohol-related cancer incidence; similarly, other studies have not found association between type of alcoholic beverage on risk of cancer-related outcomes [32, 33].

The underlying mechanisms that can explain the observed associations are relatively unknown; however, hypotheses have been generated from experimental research. It is hypothesised that polyphenols found in the wine compounds may have a role in explaining lesser harm associated with wine drinking [34]. Consuming alcohol with meals may lead to lower intestinal absorption and lower blood alcohol levels [35], while binge drinking may lead to accelerated alcohol metabolism and disrupt the antioxidant mechanisms [36]. The findings from this study may have important implications for policy and practice. These findings should be replicated and corroborated in similar large sample studies to confirm the observed association between patterns of alcohol consumption and range of adverse clinical outcomes. At an individual level, primary care and mental health professionals advising their patients on changing their drinking behaviour can give more tailored advice on various dimensions of alcohol consumption for harm reduction, based on these findings. At a policy level, there is no information available to the general population in the UK or elsewhere on health effects of different patterns of alcohol consumption [5, 6]. Our findings can help to inform future health policy advice on all dimensions of alcohol consumption patterns.

### Strengths and limitations

This study has a number of key strengths and limitations. The study had a large sample size, detailed description of various patterns of alcohol consumption, which were studied concurrently. A wide spectrum of adverse health outcomes was examined, and results were adjusted for relevant demographic, lifestyle, health-related factors and average amount of alcohol consumed. The study sample was recruited from a community setting but there is evidence of participation bias with study participants being more affluent and healthier than the average UK population [37], and therefore, it is likely that the effect sizes reported here are conservative estimates. The other major limitation of this study is the possibility of residual confounding, which is a common limitation of observational studies of harmful health effects of alcohol consumption. A systematic review of 85 studies assessing alcohol consumption risk on ischaemic heart disease found that the median number of covariates adjustment in the included studies was 9 (interquartile range 5–12) and majority of included studies failed to acknowledge the possibility of residual confounding. In our study, all-cause mortality models were adjusted for 10 covariates, liver cirrhosis models were adjusted for 11 covariates and cardiovascular events models were adjusted for 14 covariates [38]. The models in our study were adjusted for the effect of major determinants of health and well-being to minimise the risk of residual confounding [39]; however, it still remains a possibility. We only had information of alcohol consumption patterns at baseline and these patterns may have changed over the follow-up duration. While we present the findings from analysis on repeated measurements of alcohol consumption pattern during the follow-up period, that analysis was limited as follow-up data were only available for a very small subset of the study sample. We classified the type of alcoholic beverage consumed as that comprising 50% or more of the total weekly units consumed by a participant on average and conducted a sensitivity analysis with classification based on the type of alcoholic beverage consumed in the highest volume in 1 week. However, these are arbitrary methods of classifying type of alcoholic beverages and there is a possibility of overestimating or underestimating the effects of alcoholic beverage type. Finally, under reporting of alcohol consumption is common for study participants, particularly among women and binge drinkers, which may have influenced the observed results [40].

## Conclusion

Spirit and beer/cider drinking were associated with higher risk of mortality, major cardiovascular event, liver cirrhosis and accidents/self-harm when compared to red wine drinking, among regular drinkers after adjusting for alcohol amount consumed overall. Similarly, drinking alcohol without food was associated with higher mortality and cardiovascular risk compared to alcohol consumed with food when the same amount was consumed overall. Finally, spreading alcohol consumption over 3 to 4 days in a week was associated with lower mortality, cardiovascular and cirrhosis risk than consuming alcohol daily, and lower mortality and cardiovascular risk than binge drinking. The possibility of selection bias and residual confounding are important limitations of this work. These findings need to be replicated and validated in similar large-scale population studies as they have the potential to influence policy and practice advice on less harmful patterns of alcohol consumption.

## Supplementary Information

**Additional file 1.** Supplementary Analysis. Results of mediation analysis. Results of sensitivity analysis in sub-groups stratified based on amount of average alcoholic weekly units (low risk, increasing risk, high risk) and sex (male and female), alternative classification of type of alcoholic beverage, after excluding outcomes for the first two years of follow-up, and after excluding participants with poor self-rated health at baseline.

## Data Availability

The dataset used for this study will be uploaded to UK Biobank repository as per data user agreement with the UK Biobank. The dataset will be freely accessible via the repository subject to regulatory user approval from UK Biobank.

## Abbreviations

BMI: Body mass index
CI: Confidence intervals
DALY: Disability-adjusted life years
HR: Hazard ratio
LTC: Long-term conditions
MACE: Major cardiovascular events
MI: Myocardial infarction
UKB: UK Biobank
