# Supplementary material for: Association between patterns of alcohol consumption (beverage type, frequency and consumption with food) and risk of adverse health outcomes: a prospective cohort study
Source: BMC Med. 2021 Jan 12;19:8. doi: 10.1186/s12916-020-01878-2 (PMC7802201; doi:10.1186/s12916-020-01878-2)
Supplement: Supplementary file 1 — Additional file 1. Supplementary Analysis. Results of mediation analysis. Results of sensitivity analysis in sub-groups stratified based on amount of average alcoholic weekly units (low risk, increasing risk, high risk) and sex (male and female), alternative classification of type of alcoholic beverage, after excluding outcomes for the first two years of follow-up, and after excluding participants with poor self-rated health at baseline. [file 12916_2020_1878_MOESM1_ESM.docx]

**Table S1 List of self-reported long-term conditions (LTC) considered for LTC count**

| Long term condition grouping | Conditions included as reported by participants |
| --- | --- |
| 1. Painful conditions | Back pain  Joint pain  Back pain  Joint pain  Headaches (not migraine)  Sciatica  Plantar fasciitis  Carpal tunnel syndrome  Fibromyalgia  Arthritis  Shingles  Disc problem  Prolapsed disc/slipped disc  Spine arthritis/spondylitis  Ankylosing spondylitis  Back problem  Osteoarthritis  Gout  Cervical spondylosis  Trigeminal neuralgia  Disc degeneration  Trapped nerve/compressed nerve |
| 1. Hypertension | Hypertension  Essential Hypertension |
| 1. Depression | Depression  Postnatal Depression |
| 1. Asthma | Asthma |
| 1. Coronary Heart Disease | Heart attack/Myocardial Infarction  Angina |
| 1. Dyspepsia | Gastro-oesophageal reflux (GORD)/gastric reflux  Oesophagitis /Barrett's oesophagus  Gastric stomach ulcers  Gastric erosions/gastritis  Duodenal ulcer  Dyspepsia/indigestion  Hiatus hernia  Helicobacter pylori |
| 1. Diabetes | Diabetic nephropathy  Diabetic neuropathy/ulcers  Diabetes  Type 1 diabetes  Type 2 diabetes  Diabetic eye disease |
| \| 1. Thyroid disorders \| \| --- \| | Thyroid problem (not cancer)  Hyperthyroidism/thyrotoxicosis  Hypothyroidism/myxoedema  Grave’s disease  Thyroid goitre  Thyroiditis |
| 1. Connective tissue disorders | Myositis/myopathy  Systemic Lupus Erythematosus  Connective tissue disorder  Sjogrens syndrome/sicca syndrome  Dermatopolymyositis  Scleroderma/systemic sclerosis  Rheumatoid arthritis  Psoriatic arthropathy  Dermatomyositis  Polymyositis  Polymyalgia Rheumatica  Malabsorption/coeliac disease |
| 1. Chronic Obstructive Pulmonary Disease (COPD) | COPD/chronic obstructive airways disease  Emphysema/chronic bronchitis  Emphysema |
| 1. Anxiety | Anxiety/panic attacks  Nervous breakdown  Post-traumatic stress disorder  Obsessive compulsive disorder  Stress  Insomnia  Psychological/psychiatric problem |
| 1. Irritable bowel syndrome | Irritable bowel syndrome |
| 1. Alcohol problems | Alcohol dependency  Alcoholic liver disease/alcoholic cirrhosis |
| 1. Other psychoactive substance abuse | Opioid dependency  Other substance abuse/dependency |
| 1. Treated constipation | Constipation |
| 1. Stroke/Transient Ischaemic Attack (TIA) | Stroke  TIA  Subarachnoid haemorrhage  Brain haemorrhage  Ischaemic stroke |
| 1. Chronic kidney disease | Polycystic kidney  Diabetic nephropathy  Renal/kidney failure  Renal failure requiring dialysis  Renal failure not requiring dialysis  Kidney nephropathy  Immunoglobulin A (IgA) nephropathy |
| 1. Diverticular disease | Diverticular disease  Diverticulitis |
| 1. Peripheral vascular disease | Peripheral vascular disease  Leg claudication/intermittent claudication |
| 1. Heart failure | Cardiomyopathy  Hypertrophic cardiomyopathy  Heart failure/pulmonary oedema |
| 1. Prostate disorders | Prostate problem (not cancer)  Enlarged prostate  Benign prostatic hypertrophy |
| 1. Glaucoma | Glaucoma |
| 1. Epilepsy | Epilepsy |
| 1. Dementia | Dementia  Alzheimer’s disease  Cognitive impairment |
| 1. Schizophrenia/bipolar disorder | Schizophrenia  Mania/  Bipolar disorder  Manic depression |
| 1. Psoriasis/eczema | Eczema  Dermatitis  Psoriasis |
| 1. Inflammatory Bowel Disease | Inflammatory Bowel Disease  Crohn’s disease  Ulcerative colitis |
| 1. Migraine | Migraine |
| 1. Chronic sinusitis | Chronic sinusitis |
| 1. Anorexia or bulimia | Anorexia  Bulimia  Other eating disorders |
| 1. Bronchiectasis | Bronchiectasis |
| 1. Parkinson’s disease | Parkinson’s disease |
| 1. Multiple Sclerosis | Multiple Sclerosis |
| 1. Viral Hepatitis | Infective/viral hepatitis  Hepatitis B  Hepatitis C  Hepatitis D  Hepatitis E |
| 1. Chronic Liver disease | Oesophageal varices  Non infective hepatitis  Liver failure/cirrhosis  Primary biliary cirrhosis |
| 1. Osteoporosis | Osteoporosis |
| 1. Chronic fatigue syndrome | Chronic fatigue syndrome |
| 1. Endometriosis | Endometriosis |
| 1. Meniere’s disease | Meniere’s disease |
| 1. Pernicious Anaemia | Pernicious Anaemia |
| 1. Polycystic ovary | Polycystic ovary |
| 1. Atrial Fibrillation | Atrial Fibrillation |

**Table S2 Mediation Analysis: Mediating effect of different confounders on the observed association between alcohol consumption pattern and two adverse health outcomes (all-cause mortality and major adverse cardiovascular outcomes)**

|  | Total effect observed HR with 95% CI | Proportion mediated by C-reactive protein levels (95% CI) | Proportion mediated by self-rated health(95% CI) | Proportion mediated by average weekly alcohol units (95% CI) | Proportion mediated by smoking (95% CI) | Proportion mediated by Townsend score (socio-economic status) (95% CI) |
| --- | --- | --- | --- | --- | --- | --- |
| Beer or cider vs. Red wine on All-cause mortality | 1.18 (1.10-1.27); p <0.01 | 1.9% (1.2% to 3%) | 1.2% (0.6% to 3%) | 19.5% (13.5% to 25%) | 4.1% (2.6% to 7%) | 5.7% (4.2% to 8%) |
| Beer or cider vs. Red wine on MACE | 1.16 (1.05-1.27); p<0.01 | 1.4% (0.9% to 3%) | No significant mediation | 13.9% (8.8% to 25%) | 3.9% (2.5% to 7%) | 4.1% (2% to 6%) |
| Spirits vs. Red wine on All-cause mortality | 1.25 (1.14-1.38); p<0.01 | 3.2% (2.7 to 6%) | 0.6% (0.1% to 2%) | 7.2% (5% to 19.3%) | 6.1% (4.6% to 10%) | 4.8% (3.9% to 7%) |
| Spirits vs. Red wine on MACE | 1.31 (1.15-1.50); p<0.01 | 2.1% (1.2% to 4%) | 0.4% (0.1% to 1%) | 3.5% (2% to 4.8%) | 7.8% (4.4% to 13%) | 2.8% (1.2% to 4%) |
| Alcohol consumption with food vs. without food on All-cause mortality | 1.10 (1.02-1.17); p=0.01 | 1.9% (1.3% to 4%) | 1.2% (0.04% to 3%) | 24.6% (14.6% to 38%) | 4.8% (2.7% to 11%) | 9.7% (7.1% to 15%) |
| Alcohol consumption with food vs. without food on MACE | 1.08 (0.99-1.17); p=0.09 | 1.4% (0.5% to 2%) | No significant mediation | 19.7% (6.8% to 35%) | No significant mediation | 6.3% (4.2% to 18%) |
| Alcohol intake 1-2 times/week vs. 3-4 times/week on All-cause mortality | 1.09 (1.03-1.16); p<0.01 | 1.5% (1% to 3%) | 0.7% (0.04% to 3%) | No significant mediation | 10.4 % (7.4% to 15%) | 6.1% (5.1% to 10%) |
| Alcohol intake 1-2 times/week vs. 3-4 times/week on MACE | 1.14 (1.06-1.23); p<0.01 | 0.8% (0.6% to 1%) | No significant mediation | 18.6% (12% to 30%) | 6.8% (4.1% to 17%) | 2.9% (2.6% to 5%) |

HR=Hazard Ratio; CI=Confidence Intervals; MACE=Major adverse cardiovascular event.

**Table S3 Sensitivity Analysis: Type of alcohol (alternative classification) consumed and adjusted risk for poor health outcomes**

| Type of Alcohol Consumed | All-cause mortality | | MACE | | Liver Cirrhosis | | Accidents/self-harm/assaults | | New Cancer | | Alcohol cancers | |
| --- | --- | --- | --- | --- | --- | --- | --- | --- | --- | --- | --- | --- |
|  | Events=8869 (2.9%) | HR with 95% CI; p-value | Events=5246 (1.7%) | HR with 95% CI; p-value | Events=838 (0.3%) | HR with 95% CI; p-value | Events=16,818 (5.4%) | HR with 95% CI; p-value | Events= 27,543 (8.9%) | HR with 95% CI; p-value | Events=6529 (2.1%) | HR with 95% CI; p-value |
| Red wine drinkers (reference) N=90,078 (29.1%) | 2067 (2.3%) | 1 | 1067 (1.3%) | 1 | 132 (0.1%) | 1 | 4529 (5%) | 1 | 8145 (9%) | 1 | 2050 (2.3%) | 1 |
| Beer or cider drinkers N=111,199 (36%) | 3998 (3.6%) | 1.18 (1.10-1.26); p <0.01 | 2076 (2.3%) | 1.19 (1.09-1.29); p<0.01 | 421 (0.4%) | 1.28 (1.01-1.61); p=0.04 | 6321 (5.7%) | 1.09 (1.04-1.14); p<0.01 | 9959 (8.9%) | 1.00 (0.96-1.04); p=0.99 | 1644 (1.5%) | 1.02 (0.94-1.10); p=0.66 |
| White wine drinkers N=80,818 (26.1%) | 1673 (2.1%) | 1.04 (0.9-1.12); p=0.24 | 606 (1.1%) | 1.04 (0.95-1.15); p=0.37 | 142 (0.2%) | 1.24 (0.96-1.61); p=0.09 | 4264 (5.3%) | 1.05 (1.01-1.10); p=0.02 | 6786 (8.4%) | 1.01 (0.98-1.05); p=0.38 | 2149 (2.6%) | 1.03 (0.97-1.10); p=0.35 |
| Spirits drinkers N=23,454 (7.6%) | 987 (4.2%) | 1.28 (1.17-1.39); p<0.01 | 396 (2.4%) | 1.36 (1.22-1.53); p<0.01 | 122 (0.5%) | 1.60 (1.21-2.11); p<0.01 | 1477 (6.3%) | 1.07 (1.01-1.15); p=0.02 | 2342 (10%) | 1.04(0.99-1.09); p=0.16 | 602 (2.6%) | 1.03 (0.93-1.14); p=0.53 |
| Mixed N=3574 (1.2%) | 144 (4%) | 1.35 (1.12-1.62); p<0.01 | 1101 (1.6%) | 1.45 (1.15-1.84); p<0.01 | 21 (0.6%) | 1.80 (1.07-3.01); p=0.02 | 227 (6.3%) | 1.12 (0.97-1.30); p=0.12 | 311 (8.7%) | 0.96 (0.85-1.08); p=0.51 | 84 (2.3%) | 1.00 (0.79-1.28); p=0.97 |

**Legend**: Type of alcohol classified on the basis of largest amount of drink type consumed by a participant from weekly alcohol units. Alcohol new cancers= Breast, colon, rectum, larynx, liver and oesophagus. HR=Hazard Ratio; CI=Confidence Intervals; MACE=Major adverse cardiovascular event. All results adjusted for age, sex, Townsend score for socio-economic deprivation (continuous), average weekly alcohol units (continuous), alcohol consumption frequency, alcohol consumption pattern with/without meals, smoking habits, BMI, physical activity levels, number of long-term conditions, self-rated health and C-reactive protein levels at baseline. MACE events adjusted for all the above plus presence of diabetes, hypertension, systolic blood pressure and total cholesterol levels at baseline. Cirrhosis events adjusted for all of the above plus Gamma glutamyl transpeptidase levels at baseline.

**Table S4 Sensitivity Analysis: Alcohol consumption patterns and adjusted risk for poor health outcomes after excluding outcomes for first two years of follow-up (Landmark analysis)**

| *Type of Alcohol Consumed* | | | | | | |
| --- | --- | --- | --- | --- | --- | --- |
|  | All-cause mortality; HR with 95% CI; p-value | MACE  HR with 95% CI; p-value | Liver cirrhosis; HR with 95% CI; p-value | Accidents/self-harm/assaults; HR with 95% CI; p-value | All-cause cancers; HR with 95% CI; p-value | Alcohol related cancers  HR with 95% CI; p-value |
| Red wine drinkers | HR=1 | HR=1 | HR=1 | HR=1 | HR=1 | HR=1 |
| Beer or cider drinkers | HR=1.15 (1.06-1.24); p<0.01 | HR=1.16 (1.06- 1.27); p<0.01 | HR=1.39 (1.06-1.82); p=0.01 | HR=1.50 (1.15-1.95); p<0.01 | HR=1.00 (0.96-1.05); p=0.94 | HR=1.03 (0.93-1.15); p=0.49 |
| White wine drinkers | HR=1.01 (0.93-1.10); p=0.78 | HR=1.04 (0.94-1.17); p=0.38 | HR=1.06 (0.77-1.44); p=0.72 | HR=1.23 (0.90-1.68); p=0.18 | HR=1.03 (0.98-1.08); p=0.19 | HR=0.98 (0.90-1.06); p=0.62 |
| Spirits drinkers | HR=1.22 (1.10-1.35); p<0.01 | HR=1.27 (1.11-1.45); p<0.01 | HR=1.32 (0.93-1.86); p=0.11 | HR=1.68 (1.21-2.34); p<0.01 | HR=0.94 (0.87-1.00); p=0.06 | HR=0.90 (0.79-1.03); p=0.14 |
| Mixed | HR=1.06 (0.98-1.14); p=0.12 | HR=1.15 (1.05-1.26); p<0.01 | HR=1.10 (0.83-1.46); p=0.49 | HR=1.14 (0.86-1.51); p=0.35 | HR=1.02 (0.98-1.06); p=0.34 | HR=0.98 (0.90-1.07); p=0.68 |
| *Alcohol Consumed with Meals* | | | | | | |
| Yes | HR=1 | HR=1 | HR=1 | HR=1 | HR=1 | HR=1 |
| No | HR=1.10 (1.02-1.18); p<0.01 | HR=1.10 (1.01-1.20); p=0.03 | HR=0.95 (0.74-1.22); p=0.70 | HR=1.08 (0.85-1.38); p=0.51 | HR=1.03 (0.98-1.07); p=0.22 | HR=1.07 (0.97-1.17); p=0.15 |
| Mixed | HR=1.03 (0.97-1.09); p=0.36 | HR=1.04 (0.97-1.12); p=0.29 | HR=1.11 (0.89-1.38); p=0.34 | HR=1.24 (1.01-1.52); p=0.04 | HR=1.11 (1.07-1.04); p<0.01 | HR=1.13 (1.05-1.21); p<0.01 |
| *Frequency of Alcohol Intake* | | | | | | |
| 3-4 times/week | HR=1 | HR=1 | HR=1 | HR=1 | HR=1 | HR=1 |
| 1-2 times/week | HR=1.10 (1.03-1.16); p<0.01 | HR=1.15 (1.07-1.24); p<0.01 | HR=1.00 (0.79-1.28); p=0.95 | HR=0.92 (0.72-1.17); p=0.50 | HR=1.03 (0.99-1.07); p=0.10 | HR=0.99 (0.92-1.07); p=0.92 |
| Daily or almost daily | HR=1.04 (0.97-1.10); p=0.24 | HR=0.97 (0.90-1.05); p=0.48 | HR=1.61 (1.30-1.99); p<0.01 | HR=2.14 (1.75-2.63); p<0.01 | HR=0.89 (0.86-0.92); p<0.01 | HR=0.90 (0.84-0.98); p=0.01 |

**Legend:** HR=Hazard Ratio; CI=Confidence Intervals; MACE=Major adverse cardiovascular event. Alcohol new cancers= Breast, colon, rectum, larynx, liver and oesophagus. All results adjusted for age, sex, Townsend score for socio-economic deprivation (continuous), average weekly alcohol units (continuous), alcohol consumption frequency, alcohol consumption pattern with/without meals, smoking habits, BMI, physical activity levels, number of long-term conditions, self-rated health and C-reactive protein levels at baseline. MACE events adjusted for all the above plus presence of diabetes, hypertension, systolic blood pressure and total cholesterol levels at baseline. Cirrhosis events adjusted for all of the above plus Gamma glutamyl transpeptidase levels at baseline.

**Table S5 Stratified regression models (sub-group analysis). Subgroup: Excluding participants with poor self-rated health at baseline. N=300,700.**

| *Type of Alcohol Consumed* | | | | | | |
| --- | --- | --- | --- | --- | --- | --- |
|  | All-cause mortality; HR with 95% CI; p-value | MACE  HR with 95% CI; p-value | Liver cirrhosis; HR with 95% CI; p-value | Accidents/self-harm/assaults; HR with 95% CI; p-value | All-cause cancers; HR with 95% CI; p-value | Alcohol related cancers  HR with 95% CI; p-value |
| Red wine drinkers | HR=1 | HR=1 | HR=1 | HR=1 | HR=1 | HR=1 |
| Beer or cider drinkers | HR=1.18 (1.09-1.27); p<0.01 | HR=1.15 (1.04- 1.27); p<0.01 | HR=1.34 (1.03-1.74); p=0.02 | HR=1.12 (1.06-1.18); p<0.01 | HR=0.98 (0.94-1.02); p=0.40 | HR=1.03 (0.94-1.13); p=0.45 |
| White wine drinkers | HR=1.02 (0.94-1.11); p=0.58 | HR=1.06 (0.95-1.18); p=0.30 | HR=1.23 (0.91-1.65); p=0.17 | HR=1.03 (0.98-1.08); p=0.25 | HR=1.02 (0.98-1.07); p=0.22 | HR=1.01 (0.94-1.09); p=0.68 |
| Spirits drinkers | HR=1.23 (1.12-1.37); p<0.58 | HR=1.29 (1.12-1.48); p<0.01 | HR=1.57 (1.13-2.19); p<0.01 | HR=1.08 (1.01-1.16); p=0.04 | HR=1.01 (0.94-1.07); p=0.81 | HR=1.02 (0.90-1.14); p=0.77 |
| Mixed | HR=1.07 (1.01-1.15); p=0.04 | HR=1.15 (1.04-1.26); p<0.01 | HR=1.02 (0.77-1.35); p=0.90 | HR=1.01 (0.96-1.06); p=0.65 | HR=1.03 (0.99-1.06); p=0.09 | HR=0.99 (0.92-1.07); p=0.94 |
| *Alcohol Consumed with Meals* | | | | | | |
| Yes | HR=1 | HR=1 | HR=1 | HR=1 | HR=1 | HR=1 |
| No | HR=1.13 (1.06-1.21); p<0.01 | HR=1.10 (1.01-1.20); p=0.04 | HR=0.85 (0.67-1.08); p=0.20 | HR=1.04 (0.99-1.10); p=0.08 | HR=0.97 (0.93-1.01); p=0.11 | HR=0.94 (0.86-1.12); p=0.12 |
| Mixed | HR=1.04 (0.98-1.10); p=0.22 | HR=1.03 (0.96-1.11); p=0.40 | HR=0.97 (0.79-1.19); p=0.78 | HR=1.02 (0.98-1.06); p=0.32 | HR=1.01 (0.98-1.04); p=0.52 | HR=0.98 (0.93-1.04); p=0.69 |
| *Frequency of Alcohol Intake* | | | | | | |
| 3-4 times/week | HR=1 | HR=1 | HR=1 | HR=1 | HR=1 | HR=1 |
| 1-2 times/week | HR=1.10 (1.04-1.17); p=0.05 | HR=1.14 (1.06-1.23); p<0.01 | HR=1.03 (0.82-1.30); p=0.80 | HR=1.03 (0.99-1.07); p=0.15 | HR=0.98 (0.95-1.02); p=0.31 | HR=0.99 (0.93-1.05); p=0.81 |
| Daily or almost daily | HR=1.05 (0.99-1.11); p<0.01 | HR=0.97 (0.89-1.05); p=0.43 | HR=1.55 (1.27-1.91); p<0.01 | HR=1.03 (0.99-1.08); p=0.12 | HR=0.98 (0.94-1.02); p=0.49 | HR=0.99 (0.92-1.06); p=0.79 |

**Legend:** HR=Hazard Ratio; CI=Confidence Intervals; MACE=Major adverse cardiovascular event. Alcohol new cancers= Breast, colon, rectum, larynx, liver and oesophagus. All results adjusted for age, sex, Townsend score for socio-economic deprivation (continuous), average weekly alcohol units (continuous), alcohol consumption frequency, alcohol consumption pattern with/without meals, smoking habits, BMI, physical activity levels, number of long-term conditions, self-rated health and C-reactive protein levels at baseline. MACE events adjusted for all the above plus presence of diabetes, hypertension, systolic blood pressure and total cholesterol levels at baseline. Cirrhosis events adjusted for all of the above plus Gamma glutamyl transpeptidase levels at baseline.

**Table S6 Stratified regression models (sub-group analysis). Subgroup: Low Risk Alcohol consumption (Average alcohol 1-14 units/week). N=147,769.**

| *Type of Alcohol Consumed* | | | | | | |
| --- | --- | --- | --- | --- | --- | --- |
|  | All-cause mortality; HR with 95% CI; p-value | MACE  HR with 95% CI; p-value | Liver cirrhosis; HR with 95% CI; p-value | Accidents/self-harm/assaults; HR with 95% CI; p-value | All-cause cancers; HR with 95% CI; p-value | Alcohol related cancers  HR with 95% CI; p-value |
| Red wine drinkers | HR=1 | HR=1 | HR=1 | HR=1 | HR=1 | HR=1 |
| Beer or cider drinkers | HR=1.14 (1.02-1.28); p=0.02 | HR=1.16 (1.01- 1.35); p=0.04 | HR=1.20 (0.72-2.00); p=0.48 | HR=1.12 (1.03-1.22); p<0.01 | HR=0.98 (0.92-1.05); p=0.36 | HR=1.03 (0.90-1.18); p=0.72 |
| White wine drinkers | HR=0.96 (0.86-1.07); p=0.47 | HR=0.99 (0.85-1.15); p=0.96 | HR=1.24 (0.78-1.96); p=0.35 | HR=1.03 (0.97-1.11); p=0.32 | HR=1.02 (0.97-1.08); p=0.60 | HR=1.02 (0.93-1.12); p=0.58 |
| Spirits drinkers | HR=1.00 (0.86-1.15); p=0.98 | HR=1.11 (0.92-1.35); p=0.28 | HR=1.08 (0.59-1.96); p=0.80 | HR=1.13 (1.02-1.24); p=0.02 | HR=0.98 (0.92-1.05); p=0.29 | HR=1.02 (0.88-1.19); p=0.74 |
| Mixed | HR=1.03 (0.93-1.14); p=0.54 | HR=1.12 (0.98-1.27); p=0.09 | HR=0.98 (0.62-1.55); p=0.94 | HR=1.04 (0.98-1.11); p=0.20 | HR=1.03 (0.98-1.08); p=0.57 | HR=1.02 (0.92-1.12); p=0.65 |
| *Alcohol Consumed with Meals* | | | | | | |
| Yes | HR=1 | HR=1 | HR=1 | HR=1 | HR=1 | HR=1 |
| No | HR=1.07 (0.97-1.18); p=0.17 | HR=1.04 (0.90-1.19); p=0.59 | HR=1.07 (0.70-1.63); p=0.76 | HR=0.99 (0.92-1.07); p=0.90 | HR=0.93 (0.88-0.99); p=0.03 | HR=0.92 (0.82-1.03); p=0.15 |
| Mixed | HR=1.05 (0.96-1.14); p=0.26 | HR=1.08 (0.97-1.21); p=0.15 | HR=1.00 (0.69-1.45); p=0.98 | HR=0.97 (0.92-1.03); p=0.40 | HR=1.01 (0.97-1.06); p=0.48 | HR=1.01 (0.93-1.10); p=0.77 |
| *Frequency of Alcohol Intake* | | | | | | |
| 3-4 times/week | HR=1 | HR=1 | HR=1 | HR=1 | HR=1 | HR=1 |
| 1-2 times/week | HR=1.07 (0.97-1.17); p=0.17 | HR=1.07 (0.95-1.21); p=0.24 | HR=1.56 (1.03-2.36); p=0.04 | HR=1.01 (0.95-1.07); p=0.70 | HR=1.03 (0.99-1.09); p=0.09 | HR=0.97 (0.89-1.06); p=0.52 |
| Daily or almost daily | HR=1.15 (1.02-1.30); p=0.02 | HR=1.02 (0.86-1.20); p=0.82 | HR=1.77 (1.05-2.98); p=0.03 | HR=1.13 (1.04-1.22); p<0.01 | HR=1.03 (0.97-1.10); p=0.36 | HR=0.97 (0.86-1.10); p=0.69 |

**Legend:** HR=Hazard Ratio; CI=Confidence Intervals; MACE=Major adverse cardiovascular event. Alcohol new cancers= Breast, colon, rectum, larynx, liver and oesophagus. All results adjusted for age, sex, Townsend score for socio-economic deprivation (continuous), average weekly alcohol units (continuous), alcohol consumption frequency, alcohol consumption pattern with/without meals, smoking habits, BMI, physical activity levels, number of long-term conditions, self-rated health and C-reactive protein levels at baseline. MACE events adjusted for all the above plus presence of diabetes, hypertension, systolic blood pressure and total cholesterol levels at baseline. Cirrhosis events adjusted for all of the above plus Gamma glutamyl transpeptidase levels at baseline.

**Table S7** **Stratified regression models (sub-group analysis). Subgroup:** **Increasing Risk Alcohol consumption (Average alcohol intake between 15-35 units in females and 15-50 units in males). N=133,910.**

| *Type of Alcohol Consumed* | | | | | | |
| --- | --- | --- | --- | --- | --- | --- |
|  | All-cause mortality; HR with 95% CI; p-value | MACE  HR with 95% CI; p-value | Liver cirrhosis; HR with 95% CI; p-value | Accidents/self-harm/assaults; HR with 95% CI; p-value | All-cause cancers; HR with 95% CI; p-value | Alcohol related cancers  HR with 95% CI; p-value |
| Red wine drinkers | HR=1 | HR=1 | HR=1 | HR=1 | HR=1 | HR=1 |
| Beer or cider drinkers | HR=1.23 (1.10-1.37); p<0.01 | HR=1.19 (1.03- 1.37); p=0.01 | HR=1.45 (0.98-2.14); p=0.06 | HR=1.13 (1.05-1.22); p<0.01 | HR=0.97 (0.91-1.02); p=0.28 | HR=0.99 (0.86-1.14); p=0.88 |
| White wine drinkers | HR=1.08 (0.95-1.23); p=0.54 | HR=1.07 (0.89-1.28); p=0.46 | HR=1.33 (0.84-2.11); p=0.22 | HR=1.00 (0.92-1.09); p=0.99 | HR=1.02 (0.95-1.08); p=0.62 | HR=1.04 (0.92-1.17); p=0.55 |
| Spirits drinkers | HR=1.61 (1.39-1.86); p<0.01 | HR=1.42 (1.15-1.74); p<0.01 | HR=1.91 (1.15-3.16); p=0.01 | HR=1.11 (0.98-1.26); p=0.11 | HR=1.06 (0.97-1.17); p=0.20 | HR=1.10 (0.94-1.34); p=0.37 |
| Mixed | HR=1.12 (1.00-1.23); p=0.04 | HR=1.02 (0.92-1.14); p<0.01 | HR=1.04 (0.68-1.57); p=0.87 | HR=1.00 (0.93-1.08); p=0.98 | HR=1.04 (0.98-1.10); p=0.18 | HR=0.99 (0.88-1.12); p=0.88 |
| *Alcohol Consumed with Meals* | | | | | | |
| Yes | HR=1 | HR=1 | HR=1 | HR=1 | HR=1 | HR=1 |
| No | HR=1.18 (1.07-1.30); p<0.01 | HR=1.11 (0.98-1.27); p=0.11 | HR=0.64 (0.44-0.93); p=0.02 | HR=1.02 (0.94-1.10); p=0.65 | HR=0.99 (0.93-1.05); p=0.70 | HR=0.96 (0.85-1.10); p=0.59 |
| Mixed | HR=1.04 (0.96-1.13); p=0.28 | HR=1.02 (0.92-1.14); p=0.68 | HR=0.81 (0.60-1.10); p=0.18 | HR=1.00 (0.94-1.06); p=0.90 | HR=0.99 (0.95-1.04); p=0.93 | HR=0.94 (0.86-1.04); p=0.24 |
| *Frequency of Alcohol Intake* | | | | | | |
| 3-4 times/week | HR=1 | HR=1 | HR=1 | HR=1 | HR=1 | HR=1 |
| 1-2 times/week | HR=1.14 (1.04-1.25); p<0.01 | HR=1.14 (1.01-1.29); p=0.03 | HR=0.75 (0.51-1.10); p=0.13 | HR=1.12 (1.04-1.20); p<0.01 | HR=1.03 (0.97-1.09); p=0.30 | HR=1.02 (0.90-1.17); p=0.72 |
| Daily or almost daily | HR=0.99 (0.92-1.07); p=0.91 | HR=0.91 (0.82-1.01); p=0.08 | HR=1.36 (1.03-1.80); p=0.03 | HR=1.01 (0.96-1.07); p=0.65 | HR=0.96 (0.92-1.00); p=0.09 | HR=0.94 (0.86-1.03); p=0.21 |

**Legend:** HR=Hazard Ratio; CI=Confidence Intervals; MACE=Major adverse cardiovascular event. Alcohol new cancers= Breast, colon, rectum, larynx, liver and oesophagus. All results adjusted for age, sex, Townsend score for socio-economic deprivation (continuous), average weekly alcohol units (continuous), alcohol consumption frequency, alcohol consumption pattern with/without meals, smoking habits, BMI, physical activity levels, number of long-term conditions, self-rated health and C-reactive protein levels at baseline. MACE events adjusted for all the above plus presence of diabetes, hypertension, systolic blood pressure and total cholesterol levels at baseline. Cirrhosis events adjusted for all of the above plus Gamma glutamyl transpeptidase levels at baseline.

**Table S8** **Stratified regression models (sub-group analysis). Subgroup: High Risk Alcohol consumption** (**Average alcohol intake between >35 units in females and >50 units in males). N=27,444.**

| *Type of Alcohol Consumed* | | | | | | |
| --- | --- | --- | --- | --- | --- | --- |
|  | All-cause mortality; HR with 95% CI; p-value | MACE  HR with 95% CI; p-value | Liver cirrhosis; HR with 95% CI; p-value | Accidents/self-harm/assaults; HR with 95% CI; p-value | All-cause cancers; HR with 95% CI; p-value | Alcohol related cancers  HR with 95% CI; p-value |
| Red wine drinkers | HR=1 | HR=1 | HR=1 | HR=1 | HR=1 | HR=1 |
| Beer or cider drinkers | HR=1.26 (1.02-1.55); p=0.03 | HR=1.21 (0.91- 1.61); p=0.19 | HR=1.20 (0.75-1.92); p=0.44 | HR=0.96 (0.83-1.12); p=-0.62 | HR=1.08 (0.95-1.24); p=0.23 | HR=1.07 (0.82-1.41); p=0.61 |
| White wine drinkers | HR=1.15 (0.88-1.51); p=0.31 | HR=1.22 (0.84-1.79); p=0.29 | HR=0.89 (0.46-1.73); p=0.74 | HR=1.15 (0.96-1.37); p=0.12 | HR=1.10 (0.93-1.29); p=0.26 | HR=0.91 (0.68-1.22); p=0.52 |
| Spirits drinkers | HR=1.70 (1.29-2.25); p<0.01 | HR=1.67 (1.13-2.47); p<0.01 | HR=1.76 (0.99-3.12); p=0.05 | HR=1.04 (0.82-1.32); p=0.74 | HR=1.03 (0.83-1.30); p=0.75 | HR=0.76 (0.47-1.23); p=0.27 |
| Mixed | HR=1.16 (0.91-1.48); p=0.22 | HR=1.01 (0.71-1.43); p=0.95 | HR=1.40 (0.82-2.40); p=0.22 | HR=0.99 (0.83-1.18); p=0.90 | HR=1.04 (0.89-1.21); p=0.60 | HR=0.89 (0.66-1.20); p=0.46 |
| *Alcohol Consumed with Meals* | | | | | | |
| Yes | HR=1 | HR=1 | HR=1 | HR=1 | HR=1 | HR=1 |
| No | HR=1.11 (0.92-1.35); p=0.27 | HR=1.25 (0.95-1.64); p=0.10 | HR=1.37 (0.86-2.18); p=0.18 | HR=1.31 (1.12-1.53); p<0.01 | HR=0.88 (0.77-1.00); p=0.05 | HR=0.88 (0.67-1.15); p=0.33 |
| Mixed | HR=1.02 (0.85-1.22); p=0.82 | HR=1.10 (0.85-1.42); p=0.46 | HR=1.55 (0.99-2.42); p=0.06 | HR=1.29 (1.12-1.48); p<0.01 | HR=0.92 (0.83-1.04); p=0.18 | HR=0.95 (0.75-1.19); p=0.64 |
| *Frequency of Alcohol Intake* | | | | | | |
| 3-4 times/week | HR=1 | HR=1 | HR=1 | HR=1 | HR=1 | HR=1 |
| 1-2 times/week | HR=1.34 (0.95-1.90); p=0.10 | HR=2.05 (1.32-3.18); p<0.01 | HR=0.83 (0.25-2.74); p=0.76 | HR=1.08 (0.83-1.42); p=0.55 | HR=1.02 (0.76-1.36); p=0.90 | HR=0.73 (0.35-1.50); p=0.39 |
| Daily or almost daily | HR=1.09 (0.94-1.27); p=0.26 | HR=1.23 (0.99-1.54); p=0.06 | HR=1.78 (1.18-2.67); p<0.01 | HR=0.91 (0.81-1.02); p=0.11 | HR=0.99 (0.88-1.10); p=0.83 | HR=1.18 (0.94-1.50); p=0.16 |

**Legend:** HR=Hazard Ratio; CI=Confidence Intervals; MACE=Major adverse cardiovascular event. Alcohol new cancers= Breast, colon, rectum, larynx, liver and oesophagus. All results adjusted for age, sex, Townsend score for socio-economic deprivation (continuous), average weekly alcohol units (continuous), alcohol consumption frequency, alcohol consumption pattern with/without meals, smoking habits, BMI, physical activity levels, number of long-term conditions, self-rated health and C-reactive protein levels at baseline. MACE events adjusted for all the above plus presence of diabetes, hypertension, systolic blood pressure and total cholesterol levels at baseline. Cirrhosis events adjusted for all of the above plus Gamma glutamyl transpeptidase levels at baseline.

**Table S9** **Stratified regression models (sub-group analysis). Subgroup: Females. N=154,682.**

| *Type of Alcohol Consumed* | | | | | | |
| --- | --- | --- | --- | --- | --- | --- |
|  | All-cause mortality; HR with 95% CI; p-value | MACE  HR with 95% CI; p-value | Liver cirrhosis; HR with 95% CI; p-value | Accidents/self-harm/assaults; HR with 95% CI; p-value | All-cause cancers; HR with 95% CI; p-value | Alcohol related cancers  HR with 95% CI; p-value |
| Red wine drinkers | HR=1 | HR=1 | HR=1 | HR=1 | HR=1 | HR=1 |
| Beer or cider drinkers | HR=1.13 (0.98-1.32); p=0.09 | HR=1.11 (0.90- 1.38); p=0.32 | HR=1.30 (0.77-2.20); p=0.33 | HR=1.14 (1.04-1.24); p<0.01 | HR=1.01 (0.94-1.10); p=0.71 | HR=0.98 (0.86-1.12); p=0.79 |
| White wine drinkers | HR=0.96 (0.87-1.06); p=0.44 | HR=0.96 (0.83-1.11); p=0.64 | HR=1.20 (0.82-1.77); p=0.35 | HR=1.05 (0.99-1.11); p=0.08 | HR=1.02 (0.97-1.07); p=0.35 | HR=1.01 (0.94-1.09); p=0.75 |
| Spirits drinkers | HR=1.19 (1.03-1.37); p=0.01 | HR=1.20 (0.98-1.47); p=0.07 | HR=0.91 (0.52-1.57); p=0.74 | HR=1.12 (1.02-1.22); p=0.01 | HR=1.05 (0.97-1.14); p=0.20 | HR=1.05 (0.92-1.19); p=0.47 |
| Mixed | HR=1.06 (0.96-1.18); p=0.24 | HR=1.18 (1.02-1.36); p=0.03 | HR=1.52 (1.02-2.26); p=0.04 | HR=1.05 (0.99-1.12); p=0.10 | HR=1.01 (0.96-1.06); p=0.72 | HR=0.98 (0.90-1.07); p=0.70 |
| *Alcohol Consumed with Meals* | | | | | | |
| Yes | HR=1 | HR=1 | HR=1 | HR=1 | HR=1 | HR=1 |
| No | HR=1.06 (0.94-1.19); p=0.31 | HR=1.07 (0.91-1.27); p=0.41 | HR=0.76 (0.49-1.18); p=0.22 | HR=1.00 (0.93-1.07); p=0.99 | HR=0.96 (0.90-1.03); p=0.28 | HR=0.89 (0.81-0.99); p=0.03 |
| Mixed | HR=1.08 (0.99-1.18); p=0.08 | HR=1.02 (0.90-1.16); p=0.75 | HR=1.09 (0.79-1.51); p=0.57 | HR=1.02 (0.97-1.07); p=0.43 | HR=1.00 (0.96-1.05); p=0.91 | HR=1.00 (0.94-1.07); p=0.91 |
| *Frequency of Alcohol Intake* | | | | | | |
| 3-4 times/week | HR=1 | HR=1 | HR=1 | HR=1 | HR=1 | HR=1 |
| 1-2 times/week | HR=1.15 (1.04-1.26); p<0.01 | HR=1.14 (0.99-1.30); p=0.06 | HR=1.25 (0.87-1.80); p=0.22 | HR=1.04 (0.98-1.09); p=0.21 | HR=1.02 (0.97-1.07); p=0.46 | HR=0.95 (0.88-1.02); p=0.17 |
| Daily or almost daily | HR=1.00 (0.90-1.11); p=0.95 | HR=1.05 (0.90-1.21); p=0.55 | HR=1.45 (0.99-1.13); p=0.05 | HR=1.01 (0.95-1.07); p=0.79 | HR=1.02 (0.97-1.07); p=0.42 | HR=1.03 (0.95-1.12); p=0.44 |

**Legend:** HR=Hazard Ratio; CI=Confidence Intervals; MACE=Major adverse cardiovascular event. Alcohol new cancers= Breast, colon, rectum, larynx, liver and oesophagus. All results adjusted for age, sex, Townsend score for socio-economic deprivation (continuous), average weekly alcohol units (continuous), alcohol consumption frequency, alcohol consumption pattern with/without meals, smoking habits, BMI, physical activity levels, number of long-term conditions, self-rated health and C-reactive protein levels at baseline. MACE events adjusted for all the above plus presence of diabetes, hypertension, systolic blood pressure and total cholesterol levels at baseline. Cirrhosis events adjusted for all of the above plus Gamma glutamyl transpeptidase levels at baseline.

**Table S10** **Stratified regression models (sub-group analysis). Subgroup: Males. N=154,441.**

| *Type of Alcohol Consumed* | | | | | | |
| --- | --- | --- | --- | --- | --- | --- |
|  | All-cause mortality; HR with 95% CI; p-value | MACE  HR with 95% CI; p-value | Liver cirrhosis; HR with 95% CI; p-value | Accidents/self-harm/assaults; HR with 95% CI; p-value | All-cause cancers; HR with 95% CI; p-value | Alcohol related cancers  HR with 95% CI; p-value |
| Red wine drinkers | HR=1 | HR=1 | HR=1 | HR=1 | HR=1 | HR=1 |
| Beer or cider drinkers | HR=1.23 (1.13-1.34); p<0.01 | HR=1.18 (1.05- 1.31); p<0.01 | HR=1.36 (1.01-1.84); p=0.04 | HR=1.08 (1.01-1.16); p=0.02 | HR=1.00 (0.95-1.04); p=0.96 | HR=1.12 (0.98-1.29); p=0.10 |
| White wine drinkers | HR=1.17 (1.02-1.34); p=0.02 | HR=1.22 (1.02-1.44); p=0.02 | HR=1.31 (0.84-2.06); p=0.23 | HR=0.99 (0.88-1.11); p=0.84 | HR=1.02 (0.95-1.10); p=0.59 | HR=1.05 (0.83-1.31); p=0.69 |
| Spirits drinkers | HR=1.32 (1.15-1.68); p<0.01 | HR=1.34 (1.13-1.60); p<0.01 | HR=1.72 (1.16-2.57); p<0.01 | HR=1.09 (0.96-1.24); p=0.17 | HR=0.96 (0.88-1.05); p=0.38 | HR=1.08 (0.84-1.39); p=0.52 |
| Mixed | HR=1.09 (0.99-1.20); p=0.05 | HR=1.15 (1.02-1.29); p=0.02 | HR=0.90 (0.63-1.28); p=0.56 | HR=0.98 (0.91-1.06); p=0.64 | HR=1.05 (0.99-1.10); p=0.05 | HR=1.10 (0.95-1.27); p=0.19 |
| *Alcohol Consumed with Meals* | | | | | | |
| Yes | HR=1 | HR=1 | HR=1 | HR=1 | HR=1 | HR=1 |
| No | HR=1.10 (1.01-1.19); p=0.02 | HR=1.11 (1.01-1.23); p=0.04 | HR=0.97 (0.74-1.28); p=0.85 | HR=1.07 (1.01-1.15); p=0.03 | HR=0.95 (0.90-0.99); p=0.03 | HR=0.98 (0.85-1.12); p=0.77 |
| Mixed | HR=0.99 (0.92-1.06); p=0.72 | HR=1.05 (0.96-1.15); p=0.30 | HR=1.02 (0.80-1.32); p=0.84 | HR=1.01 (0.95-1.07); p=0.68 | HR=0.99 (0.95-1.03); p=0.63 | HR=0.95 (0.84-1.07); p=0.42 |
| *Frequency of Alcohol Intake* | | | | | | |
| 3-4 times/week | HR=1 | HR=1 | HR=1 | HR=1 | HR=1 | HR=1 |
| 1-2 times/week | HR=1.08 (1.01-1.16); p=0.03 | HR=1.17 (1.07-1.28); p<0.01 | HR=0.87 (0.66-1.15); p=0.34 | HR=1.04 (0.98-1.10); p=0.15 | HR=1.03 (0.99-1.08); p=0.11 | HR=1.09 (0.97-1.23); p=0.14 |
| Daily or almost daily | HR=1.06 (0.98-1.13); p=0.12 | HR=0.93 (0.85-1.02); p=0.13 | HR=1.66 (1.32-2.09); p<0.01 | HR=1.03 (0.97-1.09); p=0.28 | HR=0.96 (0.92-1.01); p=0.09 | HR=0.98 (0.87-1.10); p=0.73 |

**Legend:** HR=Hazard Ratio; CI=Confidence Intervals; MACE=Major adverse cardiovascular event. Alcohol new cancers= Breast, colon, rectum, larynx, liver and oesophagus. All results adjusted for age, sex, Townsend score for socio-economic deprivation (continuous), average weekly alcohol units (continuous), alcohol consumption frequency, alcohol consumption pattern with/without meals, smoking habits, BMI, physical activity levels, number of long-term conditions, self-rated health and C-reactive protein levels at baseline. MACE events adjusted for all the above plus presence of diabetes, hypertension, systolic blood pressure and total cholesterol levels at baseline. Cirrhosis events adjusted for all of the above plus Gamma glutamyl transpeptidase levels at baseline.

**Table S11 Participants reporting changes in Alcohol consumption pattern during follow-up period. Follow-up data available for N=15750/309144 participants**

| Type of Alcohol- changes reported from baseline | N | Frequency of Alcohol Intake-changes reported from baseline | N | Alcohol consumed with meals-changes reported from baseline | N |
| --- | --- | --- | --- | --- | --- |
| Spirits to Beer or cider drinkers | 38 | Daily or almost daily to special occasions only | 38 | Yes to no | 163 |
| Spirits to mixed drinkers | 231 | Daily or almost daily to 1-3 times/month | 43 | Yes to mixed | 1247 |
| Spirits to white wine drinkers | 54 | Daily or almost daily to 1-2/week | 299 | No to yes | 221 |
| Spirits to red wine drinkers | 81 | Daily or almost daily to 3-4 times/week | 1295 | No to mixed | 835 |
| Beer or cider to spirit drinkers | 47 | 3-4 times/week to special occasions only | 60 | Mixed to yes | 1585 |
| Beer or cider to mixed drinkers | 586 | 3-4 times/week to 1-3 times/month | 121 | Mixed to no | 965 |
| Beer or cider to white wine drinkers | 68 | 3-4 times/week to 1-2/week | 1508 |  |  |
| Beer or cider to red wine drinkers | 155 | 3-4 times/week to daily or almost daily | 731 |  |  |
| Mixed to spirits drinkers | 294 | 1-2/week to special occasions only | 253 |  |  |
| Mixed to beer or cider drinkers | 683 | 1-2/week to 1-3 times/month | 934 |  |  |
| Mixed to white wine drinkers | 743 | 1-2/week to 3-4 times/week | 971 |  |  |
| Mixed to red wine drinkers | 1417 | 1-2/week to daily or almost daily | 125 |  |  |
| White wine to spirits drinkers | 63 |  |  |  |  |
| White wine to beer or cider drinkers | 68 |  |  |  |  |
| White wine to mixed drinkers | 690 |  |  |  |  |
| White wine to red wine drinkers | 234 |  |  |  |  |
| Red wine to spirit drinkers | 108 |  |  |  |  |
| Red wine to beer or cider drinkers | 214 |  |  |  |  |
| Red wine to white wine drinkers | 308 |  |  |  |  |
| Red wine to mixed | 1575 |  |  |  |  |

**Table S12** **Extended Cox regression models with alcohol consumption patterns as time varying variables. Data available for N=15750/309144 participants**

|  | All-cause mortality; HR with 95% CI; p-value | MACE  HR with 95% CI; p-value | Liver cirrhosis; HR with 95% CI; p-value | Accidents/self-harm/assaults; HR with 95% CI; p-value | All-cause cancers; HR with 95% CI; p-value |
| --- | --- | --- | --- | --- | --- |
| *Type of Alcohol Consumed* | | | | | |
| Red wine drinkers | HR=1 | HR=1 | HR=1 | HR=1 | HR=1 |
| Beer or cider drinkers | HR=0.94 (0.6-1.46); p=0.77 | HR=0.92 (0.56- 1.51); p=0.74 | HR=10.6 (0.99-113.15); p=0.05 | HR=1.07 (0.84-1.37); p=0.59 | HR=1.05 (0.87-1.26); p=0.64 |
| White wine drinkers | HR=0.95 (0.6-1.52); p=0.84 | HR=1.14 (0.71-1.82); p=0.59 | HR=12.57 (1.52-104.01); p=0.02 | HR=0.94 (0.75-1.18); p=0.61 | HR=0.98 (0.82-1.17); p=0.82 |
| Spirits drinkers | HR=1.65 (0.98-2.77); p=0.06 | HR=1.02 (0.51-2.05); p=0.95 | HR=5.39 (0.32-90.1); p=0.24 | HR=0.8 (0.55-1.16); p=0.23 | HR=1.03 (0.79-1.35); p=0.8 |
| Mixed | HR=0.83 (0.59-1.17); p=0.28 | HR=1.13 (0.8-1.6); p=0.48 | HR=6.69 (0.83-54.1); p=0.08 | HR=0.93 (0.79-1.1); p=0.42 | HR=0.96 (0.84-1.09); p=0.5 |
| *Alcohol Consumed with Meals* | | | | | |
| Yes | HR=1 | HR=1 | HR=1 | HR=1 | HR=1 |
| No | HR=1.36 (0.94-1.95); p=0.1 | HR=0.98 (0.65-1.48); p=0.94 | HR=0.47 (0.11-2.06); p=0.32 | HR=1.06 (0.86-1.31); p=0.59 | HR=1.00 (0.85-1.17); p=0.96 |
| Mixed | HR=1.02 (0.74-1.41); p=0.91 | HR=1.02 (0.74-1.41); p=0.88 | HR=0.70 (0.25-1.99); p=0.50 | HR=1.09 (0.93-1.28); p=0.27 | HR=1.00 (0.89-1.13); p=0.99 |
| *Frequency of Alcohol Intake* | | | | | |
| 3-4 times/week | HR=1 | HR=1 | HR=1 | HR=1 | HR=1 |
| 1-2 times/week | HR=1.23 (0.86-1.76); p=0.25 | HR=1.33 (0.93-1.90); p=0.12 | HR=1.32 (0.41-4.23); p=0.65 | HR=0.99 (0.83-1.17); p=0.87 | HR=1.06 (0.93-1.21); p=0.40 |
| Daily or almost daily | HR=1.02 (0.70-1.48); p=0.93 | HR=1.04 (0.70-1.53); p=0.86 | HR=1.15 (0.33-4.01); p=0.82 | HR=0.9 (0.74-1.08); p=0.25 | HR=0.93 (0.81-1.08); p=0.34 |

HR=Hazard Ratio; CI=Confidence Intervals. All results adjusted for average weekly alcohol units (continuous), age, sex, Townsend score for socio-economic deprivation (continuous), smoking status, BMI, physical activity levels, and number of long-term conditions.
